# Stopping bosutinib reverses bosutinib-induced elevation of serum creatinine in patients with chronic myeloid leukemia

**DOI:** 10.1007/s12185-025-03954-w

**Published:** 2025-02-25

**Authors:** Maiko Abumiya, Ayano Saito, Yuki Fujioka, Masatomo Miura, Naoto Takahashi

**Affiliations:** 1 Department of Pharmacy, Akita University Hospital, Akita, Japan; 2 Department of Hematology, Nephrology and Rheumatology, Akita University Graduate School of Medicine, 1-1-1 Hondo, Akita, 010-8543 Japan; 3 Central Laboratory Division, Akita University Hospital, Akita, Japan; 4 Department of Pharmacokinetics, Akita University Graduate School of Medicine, Akita, Japan

**Keywords:** Chronic myeloid leukemia, Bosutinib, Serum creatinine, Organic cation transporter 2, Estimated glomerular filtration rate

## Abstract

Bosutinib is known to increase serum creatinine levels, and its mechanism of action is believed to involve a decrease in tubular creatinine excretion due to inhibition of tubular transporters and organic cation transporter 2. This study aimed to determine whether discontinuation of bosutinib could reverse bosutinib-induced elevation of serum creatinine levels. Serum creatinine levels were compared immediately before and after bosutinib administration and after bosutinib discontinuation in 11 patients with chronic myeloid leukemia. The median serum creatinine concentration significantly increased from 0.66 mg/dL before bosutinib to 0.76 mg/dL after bosutinib (*P* = 0.003) and decreased from 0.79 mg/dL before discontinuation of bosutinib to 0.66 mg/dL after discontinuation of bosutinib at 3 months (*P* = 0.005). This study revealed that bosutinib-induced elevation of serum creatinine, which was more pronounced in patients with the *SLC22A2* 808G/G genotype, does not indicate chronic kidney disease, but rather is simply a laboratory abnormality. If bosutinib-induced chronic kidney disease is suspected, renal function should be assessed by urinalysis and cystatin C levels to differentiate from simple elevation of serum creatinine.

## Introduction

In addition to arterial occlusive events, chronic kidney disease (CKD) is a late-onset adverse events (AEs) associated with tyrosine kinase inhibitors (TKIs) in patients with chronic myeloid leukemia (CML). The estimated glomerular filtration rate (eGFR), an indicator of CKD, is easily overlooked because it gradually declines over several years; therefore, annual evaluation is necessary. The incidence of CKD in patients treated with imatinib was 49/226 (22%), and eGFR declined approximately 3 mL/min/1.73 m^2^ annually, which is faster than the normal rate of decline with age [1].

The mechanism of TKI-induced CKD involves the combined effects of off-target actions of TKIs on PDGFR, c-KIT, SRC, and VEGFR, which are expressed in the kidney, impaired tubular cell regeneration, glomerulosclerosis, vascular endothelial damage, and atherosclerosis [2].

TKI-induced CKD is irreversible and requires early detection and prevention. In the JSH hematopoietic malignancy guidelines, TKI-induced CKD is mentioned in CML Clinical Question 4, which indicates “how to monitor late-onset side effects caused by TKIs” [3]. A systematic review and meta-analysis found that dasatinib, nilotinib, and ponatinib increased arterial occlusive events. However, no significant increase was observed following bosutinib administration [4]. Conversely, renal AEs were reported in 73/570 (13%) and 22/248 (9%) patients who received second-line or later bosutinib in phase 2 clinical trials [5] and first-line bosutinib in the BELA trial [6], respectively. The eGFR in patients receiving bosutinib declined with more patients developing grade ≥ 3b eGFR (< 45 mL/min/1.73 m^2^) with second-line or later bosutinib (139/570, 24%) and first-line bosutinib (26/248, 10%) [7]. In the BFORE study, elevated creatinine levels were reported in 18/268 (6.7%) and 22/265 (8.3%) patients in the bosutinib and imatinib groups, respectively [8]. In a phase 2 study of bosutinib in Japanese patients with newly diagnosed chronic-phase CML, 5/60 (8.3%) patients reported increased creatinine levels related to bosutinib treatment [9]. Thus, increased serum creatinine levels were observed in some patients treated with bosutinib, and bosutinib-induced CKD was irreversible and similar to that induced by imatinib.

We hypothesized that elevated bosutinib-induced serum creatinine levels are associated with the suppression of organic cation transporter 2 (OCT2) in the renal tubular epithelium and found that the increase is affected by bosutinib plasma trough concentration and 808G > T polymorphism in the *SLC22A2* gene encoding OCT2 [10]. Recently, we encountered a patient with CML who discontinued bosutinib because of breast cancer; bosutinib-induced serum creatinine elevation was reversed after cessation of bosutinib. In this study, we examined the changes in serum creatinine levels during bosutinib treatment in 11 patients with CML and showed that bosutinib-induced serum creatinine elevation was reversible after the cessation of bosutinib.

## Materials and methods

### Study design and patients

This retrospective study included patients with chronic-phase CML who received bosutinib at Akita University Hospital. This study was conducted in accordance with the principles of the Declaration of Helsinki and was approved by the Ethics Committee of the Akita University Graduate School of Medicine (no. 2235). Informed consent was obtained from all participants, and a human subject institutional review board consent form was signed.

### Sample collection and analysis of plasma bosutinib concentrations

Bosutinib was administered orally once daily in the morning. At every visit, biochemical tests, including serum creatinine tests, were performed routinely and whole blood samples were collected through venipuncture 24 ± 2 h after administration to measure the plasma concentration of bosutinib. Plasma was isolated by centrifugation at 1900 × g for 15 min and stored at − 40 ℃ until analysis. Plasma bosutinib concentrations (trough plasma concentration, C_0_) were measured using HPLC, as previously described [11].

### Identification of genotypes

DNA was extracted from peripheral blood samples using a QIAamp Blood Kit (Qiagen, Hilden, Germany) and stored at −80 °C until analysis. Genotyping procedures to identify the G and T alleles of *SLC22A2*808G > T polymorphism were carried out using PCR-restriction fragment length polymorphism (PCR–RFLP), as described by Wang et al. [12].

### Statistical analysis

Statistical analyses were conducted using SPSS software version 28.0 for Windows (IBM Corp., Armonk, NY, USA). The clinical characteristics of the patients were expressed as the number or median value (range, minimum–maximum). The daily dose, duration of bosutinib treatment, C_0_ of bosutinib, serum creatinine levels, and estimated glomerular filtration rate (eGFR) were expressed as medians and ranges. Between-group comparisons of non-parametric variables were performed using Wilcoxon signed-rank and Mann–Whitney U tests. The correlation between C_0_ and bosutinib dose was assessed using Spearman’s rank correlation coefficient. Statistical significance was set at *P* < 0.05.

## Results

### Case report

**Fig. 1:**
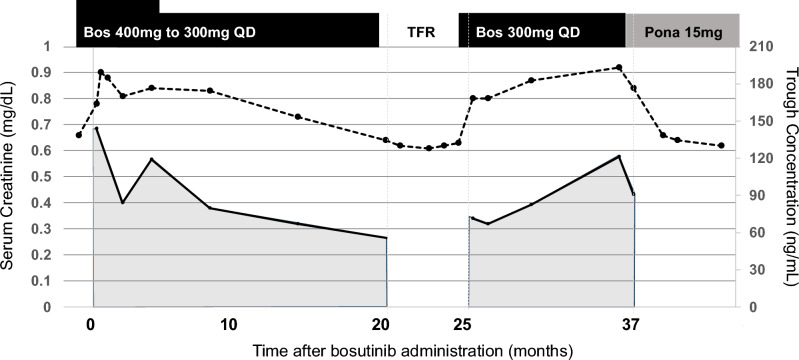
Changes in serum creatinine levels and bosutinib trough concentration in Case 1. The dotted and solid line graphs show the changes in serum creatinine levels and bosutinib blood trough concentration, respectively. TFR; treatment-free remission

A 74-year-old female with *SLC22A2* 808G/G genotype achieved MR4.5 in November 2018 and sustained DMR for 16 months with bosutinib therapy (Case1). The plasma trough bosutinib concentration during treatment was > 70 ng/mL. The patient was diagnosed with breast cancer, and bosutinib was discontinued because of chemotherapy. Although she lost MMR within 6 months of the treatment-free remission (TFR) phase, she re-achieved DMR after the re-administration of bosutinib and was switched to ponatinib for the second attempt at TFR. Serum creatinine levels increased from 0.66 mg/dL to 0.90 mg/dL after bosutinib administration and slightly decreased with the bosutinib dose reduction (Fig. 1). After discontinuation of bosutinib, the serum creatinine levels decreased from 0.77 to 0.61 mg/dL; however, they increased again because of re-administration of bosutinib. Notably, the serum creatinine levels decreased from 0.85 to 0.63 mg/dL owing to the switch to ponatinib.

### Patients’ characteristics and bosutinib treatment

This retrospective study included 11 patients who were treated with bosutinib, which was discontinued based on the TKI discontinuation criteria at the patient's request, except for Case 1. None of the patients discontinued treatment because of renal dysfunction or bosutinib-related side effects [3]. Table 1 lists the characteristics of all patients in this study. The median age at bosutinib discontinuation was 55 years (range 43–79 years). The male-to-female ratio was 4:7. The median body weight was 66 kg (range, 46–101 kg). None of the patients had serious renal or hepatic dysfunction prior to bosutinib therapy. One patient had diabetes mellitus and hypertension, whereas the other two had hypertension before bosutinib therapy, which is a risk factor for CKD. Nine and two patients harbored the *SLC22A2* 808G/G genotype and 808 T allele, respectively.

**Table 1:** Demographic and clinical characteristics and laboratory test before bosutinib therapy

Case	Age at TFR [years]	Sex	BW [kg]	CMs	*SLC22A2* 808G > T	AST [IU/L]	ALT [IU/L]	Alb [g/dL]	T.Bil [mg/dL]	sCre [mg/dL]	UP
1	74	F	49	no	G/G	23	20	4.6	0.6	0.66	–
2	52	M	101	DM/HT	G/G	17	14	4.4	0.5	0.84	–
3	46	F	68	no	G/G	16	15	4.3	0.8	0.46	–
4	67	F	66	HT	G/T	16	19	4.6	0.6	0.71	–
5	48	M	80	no	T/T	28	29	4.4	0.9	0.71	–
6	72	M	66	HT	G/G	30	32	4.7	0.8	0.81	–
7	79	M	67	no	G/G	26	23	4.1	0.9	0.84	–
8	45	F	46	no	G/G	18	24	3.9	0.9	0.45	–
9	55	F	53	no	G/G	23	34	4.3	1.7	0.43	–
10	64	F	61	no	G/G	20	16	4.2	0.5	0.49	–
11	43	F	46	no	G/G	14	17	4.2	0.6	0.54	–

*TFR* treatment-free remission, *M* male, *F* femal, *BW* body weight, *CMs* comorbidities, *DM* diabetes mellitus, *HT* hypertension, *AST* aspartate transaminase, *ALT* alanibe transaminase, *Alb* serum albumin, *T.Bil* total bilirubi, *sCre* serum creatinine, *UP* protein in urine

Table 2 presents the clinical data for bosutinib treatment. Six and five patients received bosutinib therapy as first-line treatment for newly diagnosed CML and second-line treatment for CML that was resistant or intolerant to prior TKI, respectively. The median bosutinib dose was 350 mg (range 200–500 mg). Two patients received low-dose bosutinib (200 mg). However, the median C_0_ of bosutinib for each patient did not correlate with the bosutinib dose (*P* = 0.923, Spearman’s rank correlation coefficient test). Furthermore, the median and distribution of bosutinib C_0_ was 72.0 ng/mL (49.0–113.9 ng/mL). The median duration of bosutinib treatment was 49 months (range 21–141 months).

**Table 2:** Bosutinib treatment and change of serum creatine levels by bosutinib treatment or TFR

Case	BOS treatment					sCre levels with BOS treatment			eGFR with BOS treatment		sCre levels with TFR			eGFR with TFR		
	Prior TKI (dutation, [months])	BOS dose [mg/day]	BOS duration [months]	Trough conc. [ng/mL]	TFR duratioin [months]	sCre before Bos [mg/dl]^b^	sCre at 3 M [mg/dl]	Δ sCre by Bos [mg/dl]	eGFR before Bos [ml/min/1.73m^2^]	eGFR at 3 M [ml/min/1.73m^2^]	sCre just before TFR [mg/dl]	sCre at TFR-3 M [mg/dl]	Δ sCre by TFR [mg/dl]	eGFR before TFR [ml/min/1.73m^2^]	eGFR at TFR-3 M [ml/min/1.73m^2^]	
1	no	300	21	75.6	4.1	0.66	0.90	0.24	66.2	47.1^c^	0.77	0.61	− 0.16	55.7^c^	71.6	
2	DAS (18)	400	33	72.0	3	0.84	0.89	0.05	76.4	71.7	1.00	0.81	− 0.19	62.1	78.2	
3	IM (24)	400	141	72.0	28.4^a^	0.46	0.64	0.18	121.4	84.6	0.80	0.69	− 0.11	61	71.7	
4	no	200	48	53.1	1.6	0.71	0.76	0.05	63.5	58.9^c^	0.79	0.66^e^	− 0.13	55.5^c^	67.6^e^	
5	no	400	50	76.9	35.5^a^	0.71	0.73	0.02	95.2	92.4	0.98	0.86	− 0.12	65.3	75.3	
6	no	300	41	90.3	18.8	0.81	1.35	0.54	72.5	41.4^d^	1.14	0.84	− 0.30	49.1^c^	64.3	
7	no	500	133	113.9	48.1^a^	0.84	1.21	0.37	71.8	46.9^c^	1.15	0.91	− 0.24	47.5^c^	54.8^c^	
8	NIL (18)	500	62	49.0	48.1^a^	0.45	0.55	0.10	120.0	96.4	0.57	0.50	− 0.07	89.5	102.6	
9	NIL (36)	300	50	69.0	56.3^a^	0.43	0.61	0.18	117.4	79.7	0.59	0.50	− 0.09	80.8	96.9	
10	no	200	36	104.5	63.7^a^	0.49	0.63	0.14	96.2	73.0	0.65	0.49	− 0.16	69.6	94.0	
11	IM (91)	300	45	56.0	100.7^a^	0.54	0.83	0.29	97.2	60.8	0.63	0.63	0.00	80.8	80.2	

*TKI* tyrosine kinase inhibitor, *BOS* bosutinib, *DAS* dasatinib, *IM* imatinib, *NIL* nilotinib, *conc*. concentration, *sCre* serum creatinine, Δ difference, *eGFR* estimated glomerular filtration rate, *TFR* treatment-free remission

^a^TFR continued

^b^Repost the data from Table 1

^c^CKD Grade 3a eGFR 45 ~ 59 ml/min/1.73m^2^

^d^CKD Grade 3b eGFR 30 ~ 44 ml/min/1.73m^2^

^e^At TFR-1.6 months

### Change of serum creatinine levels before and after bosutinib

Serum creatinine levels increased in all patients after bosutinib administration. Among them, four patients met the CKD grade ≥ 3a (eGFR < 60 mL/min/1.73 m^2^). Serum creatinine levels decreased in 10 patients after the discontinuation of bosutinib. Only one patient (Case 7) was classified as having CKD grade 3a, even after the discontinuation of bosutinib. A 79-year-old male with CML was treated with bosutinib for approximately 10 years and regularly took nonsteroidal anti-inflammatory drugs for knee osteoarthritis. Therefore, the patient’s eGFR declined significantly compared to the normal rate of decrease with age, and his urine was positive for protein. Tubular markers were elevated during the treatment course, which is considered true CKD grade 3a.

**Fig. 2:**
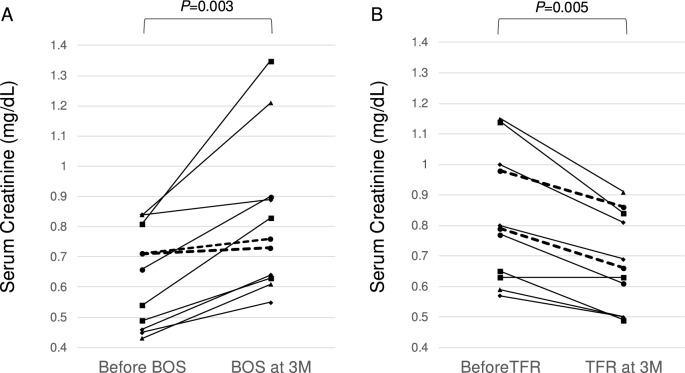
Changes in serum creatinine levels in 11 patients treated with bosutinib. **A** Changes in serum creatinine levels before and three months after bosutinib treatment. The dashed lines show the changes of two patients with 808 T allele at each point. There was a significant difference between the levels before and after bosutinib therapy (*P* = 0.003, Wilcoxon signed-rank test). **B** Changes in serum creatinine levels before and three months after cessation of bosutinib (treatment-free remission, TFR). The dashed lines show the changes in two patients with the 808 T allele at each point. There was a significant difference between the levels before and 3 months after TFR (*P* = 0.005, Wilcoxon signed-rank test)

There were significant differences in serum creatinine levels before and 3 months after bosutinib therapy (Fig. 2A). The median serum creatinine concentration was 0.63 mg/mL (0.43–0.84) and 0.75 (0.55–1.35) mg/mL before and 3 months after bosutinib therapy, respectively (*P* = 0.003, Wilcoxon signed-rank test). Notably, this change in serum creatinine levels was reversed after TFR (Fig. 2B). The median serum creatinine concentration was 0.80 (0.57–1.15) and 0.68 (0.49–0.91) mg/mL before and 3 months after TFR, respectively (*P* = 0.005, Wilcoxon signed-rank test). There was a significant difference in Δserum creatinine by bosutinib therapy between patients with the *SLC22A2* 808G/G genotype and those with the 808 T allele (0.18 vs. 0.035, *P* = 0.044, Mann–Whitney *U* test). Conversely, there was no significant difference in Δserum creatinine by TFR between both groups (− 0.16 vs. − 0.125, *P* = 0.813 Mann–Whitney *U* test).

## Discussion

This study aimed to determine whether bosutinib-induced elevation in serum creatinine levels could be reversed by discontinuation of bosutinib. The median serum creatinine concentration increased significantly after bosutinib administration and decreased after discontinuation. These findings indicate that bosutinib-induced serum creatinine elevation is a reversible laboratory abnormality and not a true renal dysfunction that causes CKD in many patients treated with bosutinib. Elevated serum creatinine levels were particularly prominent in patients with the *SLC22A2* 808G/G genotype, which was reported to be an independent factor influencing the rate of change in creatinine levels in a previous study [10]. However, all patients showed decreased creatinine levels after TFR except for Case 11. This finding indicated that the suppressive effect of OCT2 on creatinine transport in the renal tubular epithelium was relieved by the cessation of bosutinib, regardless of the *SLC22A2* 808G/G genotype or 808 T allele.

Serum creatinine is excreted from tubular epithelial cells into the urine via OCT2 and filtered out of the glomeruli [13]. OCT2 transport activity in individuals with the *SLC22A2* 808G/G genotype is significantly higher than that in individuals with the 808 T allele [12, 14, 15]. Therefore, serum creatinine values in patients with the *SLC22A2* 808 T allele tend to be higher than those with the *SLC22A2* 808G/G genotype [16, 17].Many xenobiotics increase serum creatinine levels by inhibiting active renal tubular secretion without affecting the glomerular filtration rate [18]. Bosutinib is believed to increase serum creatinine levels via OCT2 inhibition in proximal tubular epithelial cells.

Okamoro et al. revealed that the duration of TKI treatment was correlated with eGFR values at the time of TFR in 33 patients who underwent TFR [19]. However, they reported that there was no significant improvement in eGFR before or after TFR and that renal impairment was suspected to be irreversible. In this study, the first-line TKI was imatinib in 17 patients (51.5%), and the last TKI immediately before TFR was imatinib in 5 patients (15.2%) and dasatinib in 22 patients (66.2%), suggesting that more than 10 patients were switched from imatinib to dasatinib. The median duration of treatment was 4.97 years (2.56–13.89). Although eGFR declines with age, if it declines by > 1 mL/min/1.73 m^2^ annually, the influence of underlying diseases or drugs that cause renal impairment should be suspected. As imatinib-induced CKD is accompanied by impaired tubular cell regeneration, glomerulosclerosis, vascular endothelial damage, and atherosclerosis owing to off-target effects [2], it may be irreversible, as described by Okamoto et al., and an improvement in eGFR cannot be expected from imatinib TFR.

Although most cases in this study showed a decrease in serum creatinine with TFR, its increase during bosutinib treatment might not only be an apparent increase because of bosutinib's inhibition of tubular OCT2, but also owing to renal damage caused by previously used TKIs and concomitant drugs and nephrosclerosis caused by aging and complications. Therefore, it is likely that the discontinuation of bosutinib will not completely restore serum creatinine to its original level, but will only result in partial recovery in some cases.

According to the JSH guidelines [3], if CKD is suspected during TKI treatment, renal function should be assessed based on elevated serum creatinine, abnormal urinalysis, tubular damage markers, and cystatin C levels. Additionally, if CKD is diagnosed, a change in TKI or TFR is required for the treatment of underlying diseases such as hypertension, diabetes, and lipid abnormalities. In the TKI era, if the life expectancy of patients with CML does not differ from that of the general population [20], the quality of life of patients with CML must be maintained through diagnosis and intervention for the late side effects of TKI therapy, such as arterial occlusive events and CKD.

## Data Availability

Data will be made available on reasonable e-request.
